# Construction Notes

**Published:** 1894-10-27

**Authors:** 


### CONSTRUCTION NOTES.

**Bristol Royal Infirmary.**

With the proceeds of a legacy bequeathed by the late Mr. Vaughan, a new laundry has been added to the above institution, which will prove of great value to the hospital. At present washing costs £800 a-year, and by means of the addition it is computed that this expense will be curtailed by more than half.

**Wolverhampton and District Hospital for Women.**

The extension lately added to this very deserving institute gives the hospital three extra bed-rooms and two extra wards, also sitting-rooms, kitchen, pantry, and other accessories, which have become necessary as the sphere of the work has increased. Also a dispensary for out-patients has been added, and waiting rooms. Two wards have been set apart for abdominal sections. The managing committee were their own architects for this work, the contractor being Mr. J. E. Holder, 170, Great Bricklin-street, Wolverhampton.

**New Infirmary, Lewisham.**

The new infirmary for the sick poor of the Lewisham Union was opened at High Street last week. This building will accommodate 372 patients. It is arranged in two blocks of three storeys each, and a third block for the staff. The erection, which has long been felt necessary, has been delayed owing to the cost. The strictest economy has been exercised, and the total cost, including land, has been about £73,000. Messrs. W. Johnson and Co., of Wandsworth, were contractors for the main building, from designs by Messrs. A. and C. Harston, of Leadenhall-street

**Loughborough General Hospital and Dispensary,**

During the last six weeks this building has been in the hands of the workmen, and has been thoroughly renovated. The chimneys, which were not in a very sound condition, have been rebuilt of Derbyshire stone, a more durable material than the Mansfield, of which they previously were constructed. The cost was £60, and the work was carried out by Mr. Ashley. In the hall a new brass plate has been inserted in the wall opposite that commemorating the foundation of the institution by Mr. Perry Herrick, the new one being a memorial of the magnificent bequest of Mr. John Plowwright, of Loughborough, who bequeathed the residue of his estate (some £3,000) to the hospital. An additional boiler has now been provided to heat the water for the wards, and by special arrangement the furnace is used as a destructor for the dressings. Considerable improvements have been carried out in the operating theatre; the light from the skylights has been increased, and new washing appliances and cupboards for the storage of instruments have been introduced. A new operating table has been supplied by Messrs. Down Bros. All these various improvements have been carried out under the personal supervision of Mr. George Barrowcliffe, the architect, at a cost amounting to between £400 and £500.

